# Outcomes of liver transplantation of hepatoblastoma: single-center data in mainland China

**DOI:** 10.3389/fped.2025.1502761

**Published:** 2025-02-21

**Authors:** Hongting Huang, Linman Li, Jianjun Zhu, Dongwei Xu, Ping Wan, Bijun Qiu, Jiaxu Zhang, Yongkang Yang, Jie Zhao, Jianjun Zhang, Yi Luo, Mingxuan Feng, Qiang Xia

**Affiliations:** ^1^ Department of Liver Surgery, Ren Ji Hospital, Shanghai Jiao Tong University School of Medicine, Shanghai, China; ^2^ Shanghai Institute of Organ Transplantation, Shanghai, China; ^3^ Shanghai Research Center of Organ Transplantation & Immune Engineering Technology, Shanghai, China

**Keywords:** hepatoblastoma, liver transplantation, liver resection, immune suppresion, tumor metastases

## Abstract

**Objective:**

HB is the most common liver malignancy in children. Giving the rarity of the research reporting outcomes of LT for HB in China, updated long-term data are needed. The primary objective was to evaluate the outcomes of liver transplantation in HB. The secondary objective was to assess the clinical parameters that influence the outcomes of liver transplantation in HB.

**Methods:**

We retrospectively analyzed the dataset of a single-center cohort from RJ-SJTUM. Outcomes were OS and PFS. Cox proportional hazard models were used to estimate mortality adjusted HRs with 95% CIs.

**Results:**

RJ-SJTUM has accounted for 68.5% of the total cases in China since 2019. The 5-year PFS and OS rates were 63.6% and 84.6% respectively. AFP ≥13,686.5 ng/ml before LT was an independent risk factor for PFS (*P* < 0.001), and distal metastasis before LT was an independent risk factor for OS (*P* = 0.028). All patients received post-LT chemotherapy, and two patients experienced severe liver injury. Patients with localized tumor recurrence after LT had favorable outcomes if radical resection of the recurrence was achieved. Sirolimus played a role in prolonging the survival of patients with recurrent HB after LT (*P* = 0.0307).

**Conclusion:**

LT achieved favorable outcomes for patients with locally advanced hepatoblastoma. This study suggests that a judicious patient selection to exclude patients with high-risk predictors, as well as standardized postoperative management is critical in this process.

## Introduction

Hepatoblastoma (HB) is the most common primary liver cancer in infancy and childhood (1, 2), accounting for 90% of all hepatic malignancies in children younger than five years of age (3, 4). With advances in both chemotherapy and surgical treatment, including liver resection and transplantation, the prognosis of patients with HB has improved significantly (5, 6). Although the development of extreme liver resection (LR) or associating liver partition with portal vein ligation for staged hepatectomy (ALPPS) (7, 8) increase the rate of curative tumor resection in advanced HB, liver transplantation (LT) is still indicated in unresectable cases and serves as a salvage option in cases of tumor recurrence after primary liver resection (9, 10).

With the incorporation of LTs into the multidisciplinary treatment of HB, more data are required to demonstrate the performance of LT in different circumstances. Firstly, the prognostic factors for patient survival after LT require further investigation due to current small sample sizes of existing single-center cohort studies (11, 12). Second, it is questionable whether transplantation-related factors, including the timing of LT, type of graft, or immunosuppression regimen, affect HB outcomes and have important implications in clinical practice. Finally, the role of chemotherapy, including its impact on the prognosis of patients with HB and the potential risk of liver grafting, has not yet been reported.

Herein, we reviewed clinical data from RJ-SJTUM, the largest pediatric liver transplant center in mainland China, with the aim of demonstrating the current status and outcomes of LT in HB. We focused on the potential prognostic factors and several important clinical factors that are closely related to patient outcomes.

## Materials and methods

### Patient cohort and data collection

HB patients who underwent LT in RJ-SJTUM between April 2016 and August 2022 (*n* = 44, female *n* = 23, male *n* = 21) were included in the detailed analysis based on their clinical information, with follow-up until October 31, 2023, or date of death. The inclusion criteria for our study were as follows: (1) patients diagnosed with hepatoblastoma based on clinical manifestations, laboratory examinations, and pathological examinations; (2) patients who received LT during the treatment process; and (3) patients who received LT for indications other than tumor treatment, such as liver failure or surgical complications after liver resection were excluded.

Clinical information of all HB patients who underwent LT in Mainland China from January 2019 to December 2022 was collected from the China Liver Transplant Registry (CLTR).

The study protocol was approved by the Ethics Committee of RJ-SJTUM (KY2020-055). Written informed consent was obtained from all enrolled patients or their parents. All aspects of the study complied with the Declaration of Helsinki and Istanbul, and there was no unethical practice used in this study.

After adequate neoadjuvant chemotherapy, LT will be considered when the patient meets any of the following criteria, otherwise hepatectomy will be considered: (1) POST-TEXT stage IV; (2) Tumor lesions involving three main branches of the hepatic veins (left hepatic vein, middle hepatic vein, right hepatic vein) or the inferior vena cava; (3) Tumor lesions involving two main branches of the portal vein (left portal vein, right portal vein) or the main trunk of the portal vein; (4) Postoperative residual liver volume less than 20% of the standard liver volume.

All data for the clinical variables were systematically collected from our prospectively maintained database. Demographic information and clinical characteristics of all patients, including age, sex, AFP level, hepatic vein/inferior vena cava invasion, portal vein invasion, extrahepatic disease contiguous with the main liver tumor, multifocal liver tumor, tumor rupture at diagnosis, and distal metastasis, were collected before the LT operation (13).

### Treatment and follow up

Primary liver transplantation (PLT) was planned and performed in patients with unresectable tumor lesions, including tumors with multifocal lesions or major vessel involvement. Salvage liver transplantation (SLT) was defined as LT performed in patients with HB recurrence after previous LRs, indicating that the true indication for listing patients for SLT was HB recurrence rather than other indications such as biliary/portal vein complications or liver failure. For patients with major vessel involvement, a whole-liver graft was preferred to replace the involved vessels. Pre-LT chemotherapy was administered to all the patients who underwent PLT. For patients listed for SLT after LR, pre-LT chemotherapy was administered during the waiting period to prevent tumor progression. Liver transplantation was performed for curative purposes; therefore, the metastases were completely eliminated or inactivated by chemotherapy or surgery before transplantation (14). No organs from the executed patients were transplanted or included in this study.

After LT, patient follow-up included clinical parameter tests (white blood cell count, hemoglobin, platelets, albumin, alanine aminotransferase, aspartate aminotransferase, total bilirubin, creatinine, INR) every 1 week during the first three months and every month later, AFP level every three months, ultrasound tests every three months during the first year, and every six months thereafter.

All patients who underwent LT were treated with our center's standardized post-transplant immunosuppressive therapy including tacrolimus/cyclosporin, mycophenolate, and prednisone. The doses were adjusted according to serum drug concentrations and liver function assays. Glucocorticoids were discontinued within one month after LT. In the last 5 years, sirolimus was started as a part of the immunosuppression regimen approximately one month after LT for most patients.

For persistent abnormal liver function after regular adjustment for immunosuppression therapy, pathological evaluation using core-needle liver biopsy was performed to confirm the diagnosis of rejection, liver injury, or viral infection.

### Statistical analysis

Using our prospectively collected database, we analyzed patient outcomes during the current follow-up period. OS and PFS were calculated from the time of LT surgery. The survival rates of the different groups were compared using the log-rank test. Receiver operating characteristic (ROC) curves were constructed for continuous variables such as patient age, graft-to-recipient weight ratio (GRWR), intraoperative blood loss, and AFP before LT. Only the last were closely related to patient outcomes. AFP before LT was expressed as categorical variables using cut-off values. We chose the optimum cut-off to achieve the highest sum of specificity and sensitivity in the ROC curves. Univariate and multivariate Cox regression analyses were used to examine associations between patient demographics, clinical characteristics, OS, and PFS. In the multivariable Cox regression analysis, we included all factors with *P* ≤ 0.1 in the univariate analysis. The *P* values and hazard ratios (HR) of all factors in univariate Cox analysis were determined using the enter method, whereas the forward LR method was used in multivariate analysis. Unless otherwise stated, all analyses were performed with significance set at *P* < 0.05. Data collection and analysis were performed using SPSS 23.0 (Statistical Package for the Social Sciences, SPSS Inc., Chicago, IL, USA) and GraphPad Prism 8.4.0.

## Results

### Study population

**Figure 1:**
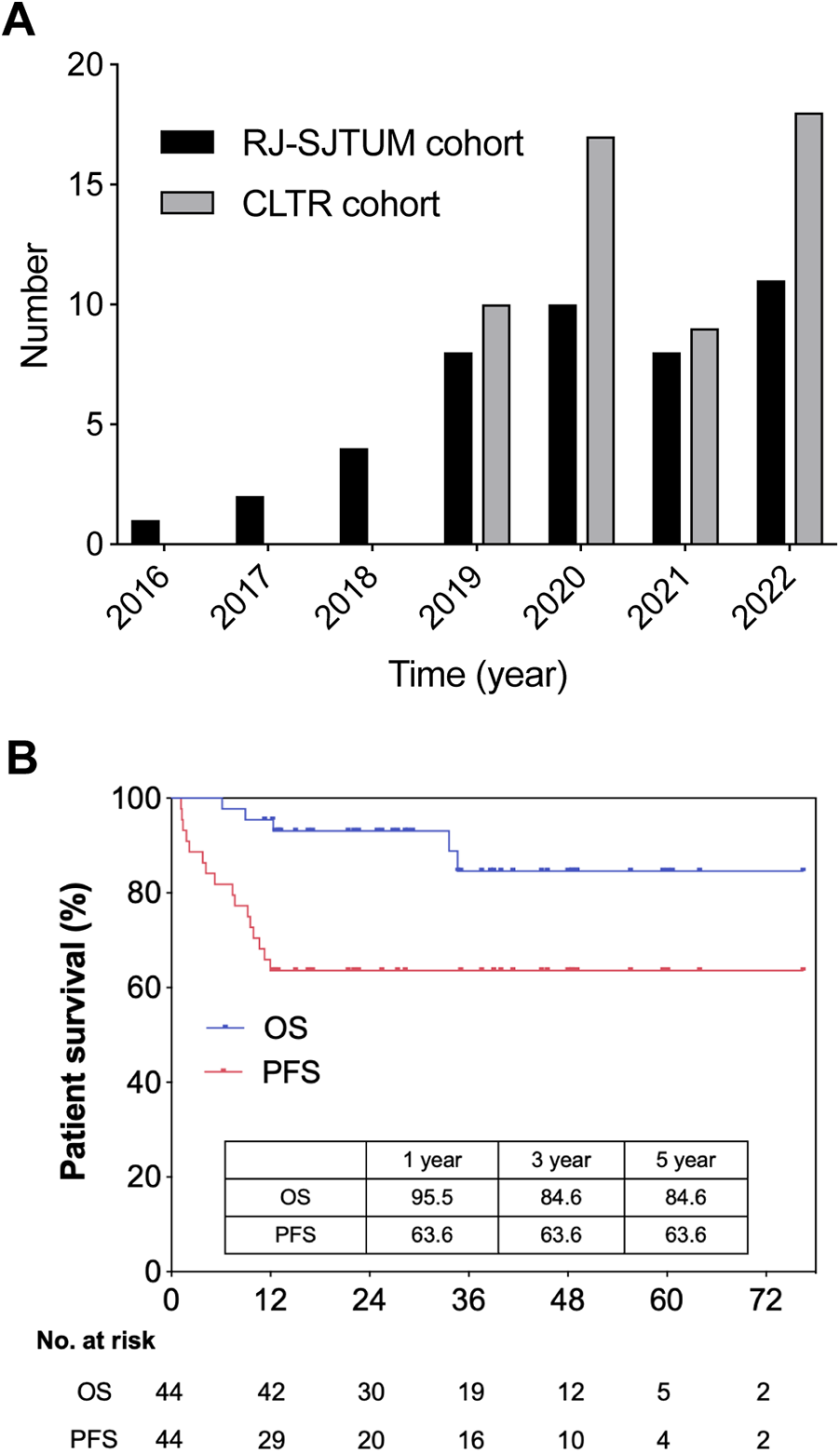
National and single center study population. **(A)** Number of pediatric LT for unresectable hepatoblastoma in RJ-SJTUM cohort and CLTR cohort. **(B)** Overall survival and progression free survival of LT group (*n* = 44) in RJ-SJTUM.

Between 2016 and 2022, 44 patients with HB underwent LT in RJ-SJTUM. The median follow-up duration was 35.0 (95% CI: 23.3–46.7) months. According to data from the CLTR (the official national liver transplant registry in Mainland China), between 2019 and 2022, 54 patients with HB underwent LT in Mainland China, of which 37 (68.5%) were from RJ-SJTUM (Figure 1A).

### Profiles and outcomes of HB patients underwent LT

As mentioned above, Supplementary Table S1 summarized the patient and tumor characteristics of the RJ-SJTUM cohort. Notably, deceased donors were the key source for HB patients (77.3%) and the whole liver was the first choice (68.2%). Living-donor transplantation was not widely performed for HB in our cohort (22.7%). The OS rates at 1, 3, and 5 years after LT were 95.5%, 84.6%, and 84.6%, respectively, and the PFS rates at 1, 3, and 5 years were 63.6%, 63.6%, and 63.6% (Figure 1B). According to the CLTR follow-up data, the 3-year OS was 82.22% and the 5-year PFS was 63.64% in Mainland China.

### Independent prognostic predictors of PFS

We performed univariate and multivariate analyses in the LT cohort (*n* = 44) to identify the independent risk predictors of PFS after LT (Table 1). In univariate analysis, two factors were identified: patient with distal metastasis before LT (HR: 3.685, 95% CI: 1.269–10.705, *P* = 0.017) and patient with serum AFP ≥13,686.5 ng/ml before LT (after pre-transplantation chemotherapy) (HR: 6.825, 95% CI: 2.350–19.825, *P* < 0.001). In multivariate analysis, AFP ≥13,686.5 ng/ml before LT (HR: 6.825, 95% CI: 2.350–19.825, *P* < 0.001) was the only independent risk factor for PFS.

**Table 1:** Univariable and multivariable Cox regression for the PFS of patients underwent LT.

	Event-free survival			
	Univariate		Multivariate	
	HR (95% CI)	*p*	HR (95% CI)	*p*
Sex				
Female (*n* = 23)	Ref			
Male (*n* = 21)	1.691 (0.629–4.546)	0.298		
PRETEXT stage				
I & II (*n* = 15)	Ref			
III & IV (*n* = 29)	0.794 (0.288–2.187)	0.655		
V				
No (*n* = 39)	Ref			
Yes (*n* = 5)	0.427 (0.056–3.233)	0.410		
P				
No (*n* = 32)	Ref			
Single brunch (*n* = 3)	0.997 (0.129–7.725)	0.997		
Double brunch (*n* = 2)	3.215 (0.704–14.677)	0.132		
Trunk (*n* = 7)	0.802 (0.178–3.621)	0.775		
E				
No (*n* = 40)	Ref			
Yes (*n* = 4)	1.703 (0.386–7.513)	0.482		
F				
No (*n* = 17)	Ref			
Yes (*n* = 27)	1.579 (0.548–4.546)	0.397		
R				
No (*n* = 43)	Ref			
Yes (*n* = 1)	1.000 (0.000–514,646.247)	1.000		
M				
No (*n* = 37)	Ref			
Yes (*n* = 7)	3.685 (1.269–10.705)	0.017		
AFP before LT ≥13,686.5 ng/ml				
No (*n* = 29)	Ref		Ref	
Yes (*n* = 15)	6.825 (2.350–19.825)	0.000	6.825 (2.350–19.825)	0.000
Primary vs. Salvage				
Primary (*n* = 20)	Ref			
Salvage (*n* = 24)	0.805 (0.302–2.145)	0.664		
Graft type				
Whole (*n* = 30)	Ref			
Partial (*n* = 14)	1.398 (0.508–3.850)	0.517		
Donor type				
Living (*n* = 10)	Ref			
Deceased (*n* = 34)	0.917 (0.296–2.846)	0.881		
Sirolimus use				
Yes (*n* = 31)	Ref			
No (*n* = 13)	0.735 (0.237–2.282)	0.594		
Bile duct anastomosis				
Duct-to-duct (*n* = 27)	Ref			
Cholangioenteric (*n* = 17)	0.998 (0.363–2.746)	0.997		

V, hepatic vein/inferior vena cava tumor invasion; P, portal vein tumor invasion; E, extrahepatic disease contiguous with the main liver tumor; F, multifocal liver tumor; R, tumor rupture at diagnosis; M, distal metastasis; AFP, α-fetoprotein; LT, liver transplantation.

**Figure 2:**
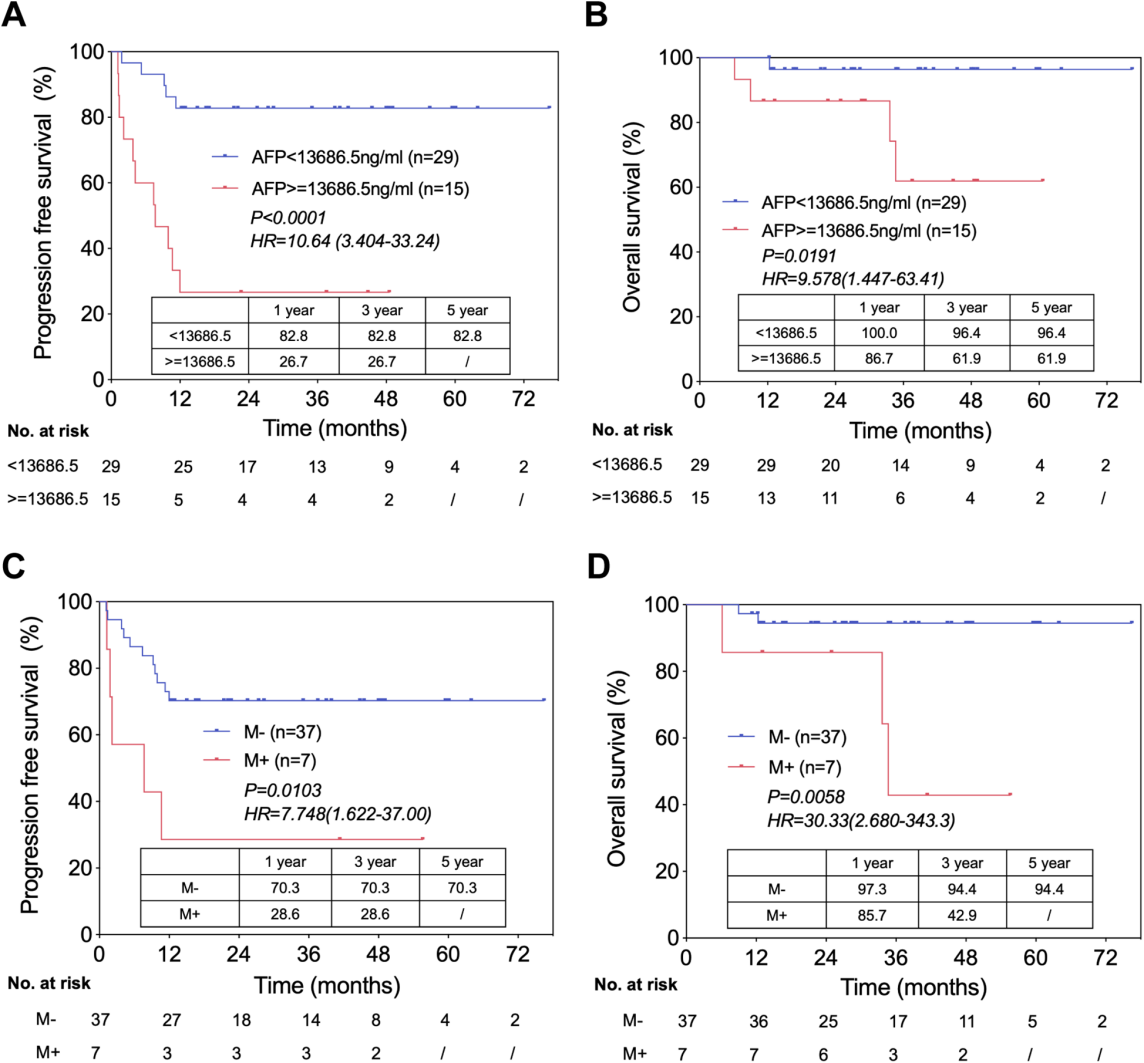
Independent risk factors of prognosis in HB patients underwent LT. **(A)** Progression free survival in patients with AFP ratio <13,686.5 ng/ml versus with AFP ratio ≥13,686.5 ng/ml, from the time of LT. **(B)** Overall survival in patients with AFP <13,686.5 ng/ml versus with AFP ≥13,686.5 ng/ml, from the time of LT. **(C)** Progression free survival in patients with distal metastasis (before LT) versus without distal metastasis, from the time of LT. **(D)** Overall survival in patients with distal metastasis (before LT) versus without distal metastasis, from the time of LT.

Survival analysis showed that PFS in HB patients with AFP <13,686.5 ng/ml was significantly higher than that in those with AFP ≥13,686.5 ng/ml (1/3/5 year OS: 82.8%/82.8%/82.8% vs. 26.7%/26.7%/-; *P* < 0.0001) (Figure 2A). As for OS, patients with AFP ≥13,686.5 ng/ml also showed poorer prognosis (1/3/5 year OS: 100.0%/96.4%/96.4% vs. 86.7%/61.9%/61.9%; *P* = 0.0191) (Figure 2B).

### Independent prognostic predictors of OS

Similarly, we attempted to identify key predictors of OS in the LT cohort using univariate and multivariate analyses (Table 2). In univariate analysis, two factors were identified as credible candidates: distal metastasis before LT (HR, 8.246; 95% CI: 1.374–49.481, *P* = 0.021) and not using sirolimus (HR, 7.948; 95% CI: 0.872–72.436, *P* = 0.066). In multivariate analysis, distal metastasis before LT (HR, 7.513; 95% CI: 1.239–45.553, *P* = 0.028) was the only independent predictor of OS in HB patients who underwent LT.

**Table 2:** Univariable and multivariable Cox regression for the OS of patients underwent LT.

	Event-free survival			
	Univariate		Multivariate	
	HR (95% CI)	*p*	HR (95% CI)	*p*
Sex				
Female (*n* = 23)	Ref			
Male (*n* = 21)	1.905 (0.318–11.425)	0.481		
PRETEXT stage				
I & II (*n* = 15)	Ref			
III & IV (*n* = 29)	0.832 (0.139–4.984)	0.841		
V				
No (*n* = 39)	Ref			
Yes (*n* = 5)	2.307 (0.253–21.039)	0.458		
P				
No (*n* = 32)	Ref	0.994		
Single brunch (*n* = 3)	0.000 (0.000-.)	0.097		
Double brunch (*n* = 2)	7.658 (0.694–84.493)	0.987		
Trunk (*n* = 7)	0.802 (0.000-.)			
E				
No (*n* = 40)	Ref			
Yes (*n* = 4)	0.043 (0.000–2,217,087.24)	0.729		
F				
No (*n* = 17)	Ref			
Yes (*n* = 27)	54.435 (0.037–80,472.843)	0.283		
R				
No (*n* = 43)	Ref			
Yes (*n* = 1)	0.048 (0.000-.)	0.885		
M				
No (*n* = 37)	Ref		Ref	
Yes (*n* = 7)	8.246 (1.374–49.481)	0.021	7.513 (1.239–45.553)	0.028
AFP before LT ≥6,208.0 ng/ml				
No (*n* = 29)	Ref			
Yes (*n* = 15)	68.676 (0.052–91,571.061)	0.249		
Primary vs. Salvage				
Primary (*n* = 20)	Ref			
Salvage (*n* = 24)	1.276 (0.213–7.656)	0.790		
Graft type				
Whole (*n* = 30)	Ref			
Partial (*n* = 14)	3.099 (0.514–18.675)	0.217		
Donor type				
Living (*n* = 10)	Ref			
Deceased (*n* = 34)	0.482 (0.080–2.902)	0.426		
Sirolimus use				
Yes (*n* = 31)	Ref		Ref	
No (*n* = 13)	7.948 (0.872–72.436)	0.066	7.127 (0.786–64.654)	0.081
Bile duct anastomosis				
Duct-to-duct (*n* = 27)	Ref			
Cholangioenteric (*n* = 17)	1.960 (0.316–12.138)	0.470		

V, hepatic vein/inferior vena cava tumor invasion; P, portal vein tumor invasion; E, extrahepatic disease contiguous with the main liver tumor; F, multifocal liver tumor; R, tumor rupture at diagnosis; M, distal metastasis; AFP, α-fetoprotein; LT, liver transplantation.

We also compared the outcomes of patients without distal metastasis before LT vs. those with distal metastasis and found that the latter had significantly lower PFS (1/3/5 year OS: 70.3%/70.3%/70.3% vs. 28.6%/28.6%/-; *P* = 0.0103) (Figure 2C) and OS (1/3/5 year OS: 97.3%/94.4%/94.4% vs. 85.7%/42.9%/-; *P* = 0.0058) (Figure 2D).

### Clinical decision-making in HB patients underwent LT

We focused on clinical decision-making factors that were closely related to LT, including primary vs. salvage LT, graft type (whole liver vs. partial liver), donor type (living vs. deceased), and bile duct anastomosis (duct-to-duct vs. cholangioenteric). None of these factors affected PFS (Table 1) or OS (Table 2) in patients with HB who underwent LT.

### Postoperative complications

Surgical complications occurred in three patients, including hepatic artery thrombosis (HAT) in two and biliary complications in one. One case of HAT occurred on the 1st day after LT, and the patient underwent a repeat surgery. The other case occurred on the 7th day after LT, and the patient received conservative treatment. Biliary stenosis occurred in one patient 1 month after LT. The patient underwent percutaneous transhepatic cholangial drainage (PTCD) and recovered 3 months later.

All the patients in our cohort received post-LT chemotherapy. Two patients had severe life-threatening liver injury caused by chemotoxicity. Notably, both patients were treated with ifosfamide, carboplatin, and etoposide (ICE) regimens. One patient recovered after eight months, and the other patient was listed for re-transplantation. In addition, one patient developed symptomatic Epstein-Barr virus infection.

Although we administered relatively mild immunosuppressive regimens to prevent tumor recurrence, we did not detect any severe graft rejection events that required liver biopsy in our cohort.

### Demographics and outcomes of recurrent patients after LT

In our study, sixteen patients experienced tumor recurrence after LT, of which five died. All patients relapsed within 1 year after LT. Table 3 summarized the detailed demographics and tumor characteristics of these patients. The sites of recurrence were the lung in eleven patients, the liver in three patients, the lung/brain in one patient, and the lung/liver in one patient. Patients with localized tumor recurrence (mostly in the lung) had favorable prognoses with pulmonary surgeries to completely eliminate recurrent tumors. However, the prognosis of patients with diffuse tumor recurrence (in the liver, lung, and brain) is dismal.

**Table 3:** Demographics and tumor characteristics of recurrent patients after LT.

Patient	Sex	Age at LT (m)	M	V	P	E	F	R	Pre-LT AFP (ng/ml)	Primary or Salvage	Donor type	GRWR (%)	Sirolimus use	Recurrence site	Outcome
1	Female	12.6	−	−	−	−	−	−	44,284	Primary	Living	3.43	Yes	Lung	Alive
2	Female	37.9	−	−	−	−	+	−	44,870	Primary	Deceased	5.67	Yes	Lung	Alive
3	Male	59.4	−	−	−	−	−	−	1,618	Salvage	Deceased	1.75	Yes	Lung	Alive
4	Male	26.7	−	−	−	−	−	−	8,648	Salvage	Deceased	3.05	Yes	Lung	Alive
5	Male	20.5	−	−	−	−	+	−	14,471	Primary	Deceased	3.76	Yes	Lung	Alive
6	Female	63.2	−	−	−	−	+	−	60,500	Primary	Deceased	1.94	Yes	Lung	Alive
7	Male	110	−	−	+	−	−	−	343	Salvage	Deceased	2.13	Yes	Liver	Alive
8	Female	96.2	+	−	+	+	+	−	60,500	Primary	Deceased	2.45	Yes	Lung	Alive
9	Female	56.8	−	−	+	−	+	−	60,500	Salvage	Living	2.31	Yes	Lung	Alive
10	Male	51.7	+	−	+	+	−	−	3,766	Salvage	Deceased	2.63	Yes	Lung	Alive
11	Male	111	−	−	−	−	+	+	38,572	Primary	Deceased	1.62	Yes	Lung	Alive
12	Female	80.9	+	+	−	−	+	−	60,500	Primary	Deceased	2.91	No	Liver	Dead
13	Male	83.2	+	−	−	−	+	−	60,500	Primary	Living	1.00	No	Liver	Dead
14	Male	37.1	+	−	−	−	+	−	13,873	Salvage	Deceased	3.14	Yes	Lung/brain	Dead
15	Male	29.9	−	−	−	−	+	−	25,277	Salvage	Deceased	2.52	No	Liver	Dead
16	Female	24.5	−	−	+	−	+	−	6,700	Salvage	Living	2.40	No	Lung/liver	Dead

V, hepatic vein/inferior vena cava tumor invasion; P, portal vein tumor invasion; E, extrahepatic disease contiguous with the main liver tumor; F, multifocal liver tumor; R, tumor rupture at diagnosis; M, distal metastasis; AFP, α-fetoprotein; LT, liver transplantation.

**Figure 3:**
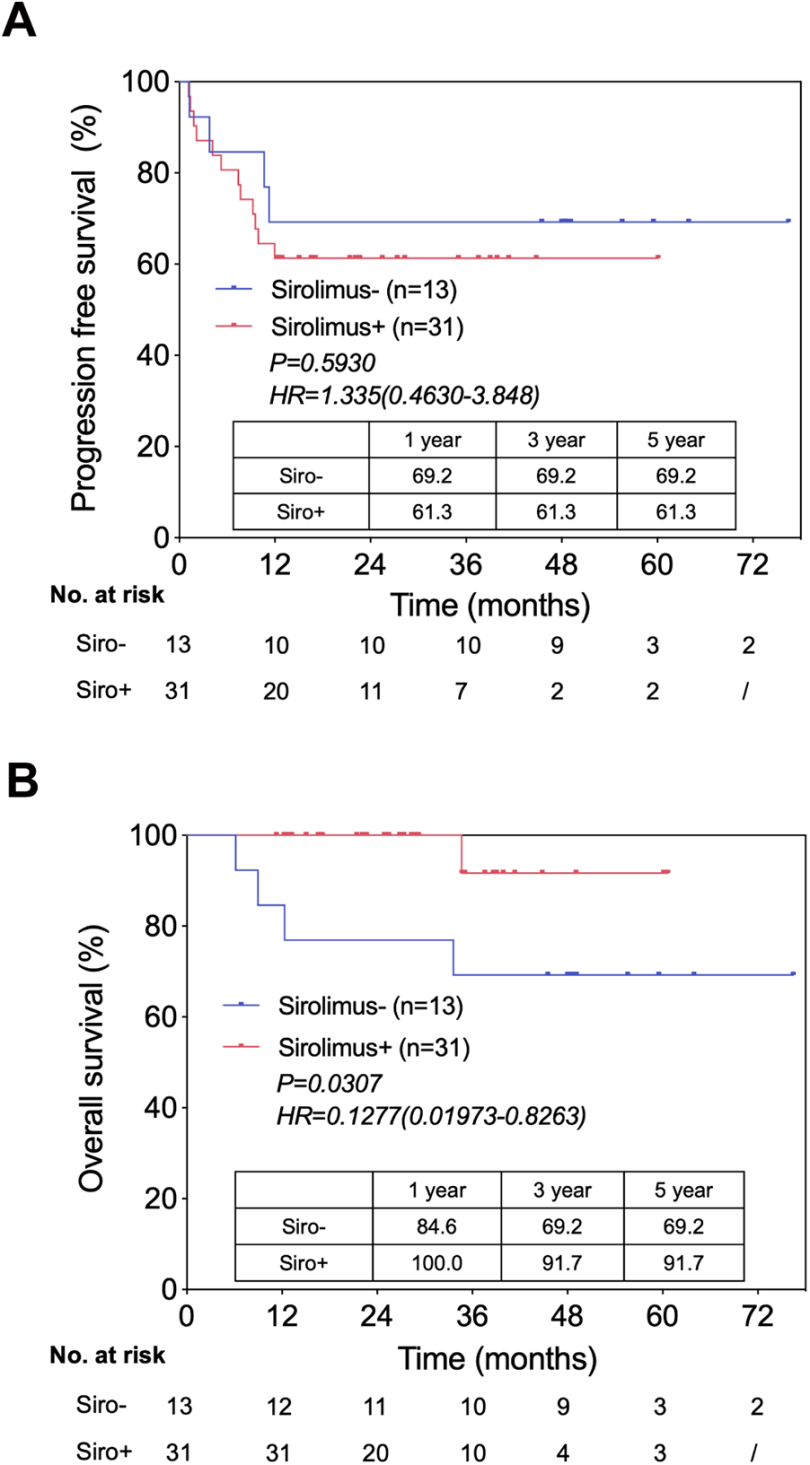
Patient survival by sirolimus usage after LT. **(A)** Progression free survival in patients use sirolimus versus not use sirolimus, from the time of LT. **(B)** Overall survival in patients use sirolimus versus not use sirolimus, from the time of LT.

mTOR inhibitors have been shown to be immunosuppressants with anti-tumor activity. In the last 5 years, we started using sirolimus one month after LT in all patients who could tolerate it. We compared the PFS and OS between patients treated with and without sirolimus after LT (Figures 3A,B). Although the results showed no statistical difference in PFS (Figure 3A), sirolimus appeared to prolong the OS (Figure 3B). We also found that of the five patients who died from tumor recurrence, four (80%) did not use sirolimus after LT. Interestingly, all eleven patients who survived tumor recurrence used sirolimus as a part of the immunosuppression regimen after LT (Table 3). Our results require further validation because of the short follow-up period and the small sample size. The relationship between sirolimus usage and HB patient prognosis after LT should be further explored in future studies, especially in patients with recurrence.

## Discussion

The present study is one of the few to focus on the status and outcomes of patients with HB who underwent LT in China. Our analysis of national data suggested that although the survival outcomes of the patients were comparable to those of the US (15, 16), the number and proportion of LT for HB were still relatively low in China (3, 16). Considering the shortage of pediatric donors, listing priorities for patients with advanced HB is recommended. Surgical innovation in complicated cases and living donor liver transplantation (LDLT) in selected cases have provided additional LT opportunities. In addition, considering liver injury caused by chemotoxicity, the use of chemotherapy after LT requires further evaluation. As one beginning of these efforts, we analyzed the RJ-SJTUM cohort, the largest cohort in China, to provide valuable results related to HB clinical practice for both China and the entire community.

We identified 2 risk factors for survival prognosis that may be applicable to the LT cohort. The first predictor was patient serum AFP level prior to LT. It has been reported that change in serum AFP level could be used to assess HB patient responsiveness to chemotherapy, and the log-fold change in AFP during chemotherapy was a predictive factor for survival in HB patients underwent LT (12, 16–18). Here we used the absolute AFP value for the following three reasons. First, the rate of change in the AFP level was determined by comparing the values before surgery and at diagnosis in previous studies (12, 16). Since the clinical courses were very complex and changeable in HB patients underwent LT, especially for those underwent SLT, we considered that the rate of change in AFP was not suitable for this kind of patients. Second, the log-fold change in AFP reflects patient responsiveness to chemotherapy, while it cannot indicate the total tumor cell burden before LT. Third, for most patients, their serum AFP values at diagnosis exceeded the upper limit of the value that the instrument can measure. Thus, it was difficult to calculate the exact values of the change rates. Another predictor of OS is distal metastasis before LT. In the CHIC study, distal metastasis at diagnosis was the strongest risk factor for poor prognosis in the LR cohort (15). Although the metastases are completely eliminated by chemotherapy or surgery before LT, patients with distal metastases before LT usually harbor more aggressive tumors (19, 20). In addition, undetectable micro-metastases might still exist before LT and resurge upon immunosuppression after LT. Interestingly, we did not identify any other predictors of survival, such as multifocal liver tumor or vessel involvement, which supports the hypothesis that LT may be the first choice for patients with extensive tumor involvement without active extrahepatic lesions.

Clinical decision-making factors, such as LT type, donor type, and bile duct anastomosis, did not affect PFS or OS in HB patients who underwent LT. Although it has been historically reported that SLT had significant lower survival (about 30%–40%) than PLT (9), recent studies have suggested that the long-term survival of SLT in HB was similar to that of PLT (16, 17). Consistently, we also found no significant difference in PFS and OS between the PLT and SLT groups, in which there were no differences in clinical characteristics (except for multifocal liver tumors and intraoperative blood loss) (data not shown). These results suggest that the SLT could be a reasonable lifesaving option in the current decision-making process.

The grafts used in our study comprised both whole and partial grafts. In our cohort, the HAT risk was higher in patients who received whole liver grafts. However, for patients with major vessel involvement, whole liver grafts were preferred to replace the involved vessels. Owing to concerns regarding tumor recurrence, grafts from living donors are usually less considered in high-risk patients. However, LDLT is advantageous in terms of shorter waiting time, better timing and preparation for surgery, and better graft quality. LDLT has yielded good outcomes for hepatic malignancies when deceased donors are scarce (21). The adoption of LDLT in HB settings warrants re-evaluation to increase the feasibility of transplantation. Nevertheless, patient selection in this setting is important for guaranteeing good outcomes. According to our recommendation, HB patients without major vessel involvement, without distal metastasis before LT, and with AFP <13,686.5 ng/ml before LT could be ideal candidates for LDLT.

Regarding postoperative complications, we report, for the first time, the occurrence of post-chemotherapy liver injury after LT in patients with HB. In our cohort, 2 cases occurred in children treated with the ICE regimen, which was previously recommended for HB patients in the high-risk group (22). This suggests that, in cases of unstable organ status in the early stages after LT, chemotherapy drugs with hepatotoxicity should be used with caution, and it is necessary to closely monitor liver function during postoperative chemotherapy. In addition, rejection was uncommon in children who underwent LT for HB, which was ascribed to the combined immunosuppressive effects of anti-rejection and anti-tumor therapy. This result was consistent with those of previous studies (16, 23) and suggested that a less intensive modified immunosuppressive regimen could be used in these patients to prevent side effects, especially infection and renal injury. Further studies are required to validate these observations.

It is important to analyze the disease processes in patients with tumor recurrence after LT. Of the 16 patients with localized distal metastases recurrence in the lungs, all patients had their metastases completely regressed by chemotherapy or removed by surgical resection, and were currently alive without tumor relapse. These results were consistent with those of previous studies and showed that children with completely eliminated pulmonary metastases post-LT demonstrated favorable long-term survival (16, 19). Accordingly, LT seemed to have a wider indication and showed good outcomes in HB, which makes it a good choice for patients with advanced high-risk HB. This superior role of LT in HB was different from that in adult hepatocellular carcinoma (HCC), in which LT was only reserved for patients within strict criteria owing to poor prognosis in advanced disease and scarcity of organs (24).

Sirolimus, an mTOR inhibitor, is an immunosuppressant with activities that can restrain tumor growth, including anti-proliferative (25), anti-angiogenic (26) and pro-immunogenic effects (27). It has been reported that sirolimus can improve the prognosis of liver recipients with HCC (28). Although the use of sirolimus for immunosuppression following LT did not affect the PFS of HB patients, which was consistent with previous research (16), we noticed that it appeared to prolong the OS. Furthermore, in the recurrent group, lack of sirolimus was closely associated with poor prognosis. Owing to the small number of subjects, further investigations and larger cohorts are needed to decipher the relationship between sirolimus and HB patient outcomes after LT.

Although our current results supported the role of LT in HB, the clinical-decision debate on LT vs. extended hepatic resection or complicated resection for advanced or high-risk HB cases still worth further discussion. Although technically challenging, extended hepatic resection could achieve good outcome, which is numerically comparable with LT (29, 30). Considering the disadvantages of LT such as organ shortage, as well as adverse effects of long-term immunosuppression after LT, the hepatic resection should still be considered prior to LT if the resection could be performed safely in centers with high expertise of liver surgery. Moreover, in contrasted with the early data suggesting significant advantage of primary LT compared with salvage LT (9), several recent studies demonstrated similar survival outcome (16, 17), providing evidence supporting hepatectomy first and LT as salvage therapy. Nevertheless, high level evidence from big sized clinical study is still needed for better selection of LT or hepatectomy for high-risk HB cases.

Our study has some limitations. First, the sample size was small and the follow-up period was relatively short. Second, several valuable perioperative and histopathological variables were not included in this study, such as microscopic or macroscopic vascular invasion on histopathology, tumor histology, detailed chemotherapy, and response to chemotherapy. Finally, our study was based on the analysis of prospectively collected data over a period of time during which surgical skills, chemotherapy modalities, policies, access to liver transplantation, and perioperative management might have evolved.

In conclusion, we confirmed that LT achieved good outcomes in HB patients. A serum AFP ≥13,686.5 ng/ml before LT was associated with worse PFS, and distal metastasis before LT was associated with worse OS after LT. Patients with localized tumor recurrence after LT showed favorable outcomes, and the use of sirolimus might play a role in prolonging survival in patients with recurrent HB after LT. These results could contribute to better patient selection for different surgical treatments and highlight the role of LT in high-risk patients. We advocate coordinated efforts to solidify the role of LT in advanced HB and to standardize the comprehensive treatment of this subgroup of patients.

## Data Availability

The original contributions presented in the study are included in the article/Supplementary Material, further inquiries can be directed to the corresponding author.
