# Supplementary material for: Outcomes of liver transplantation of hepatoblastoma: single-center data in mainland China
Source: Front Pediatr. 2025 Feb 21;13:1502761. doi: 10.3389/fped.2025.1502761 (PMC11885259; doi:10.3389/fped.2025.1502761)
Supplement: Supplementary file 1 [file Supplementaryfile1.docx]

**TableS1. Demographics and tumor characteristics of HB patients underwent LT**

|  | HB patient underwent LT (n= 44) |
| --- | --- |
| Sex   - Male - Female | 21 (47.7)  23 (42.3) |
| Age (m) | 62.4 (12.7-149.7) |
| PRETEXT stage   - I&II - III&IV | 15 (34.1)  29 (65.9) |
| V+ | 5 (11.4) |
| P+ | 12 (27.3) |
| E+ | 4 (9.1) |
| F+ | 27 (61.4) |
| R+ | 1 (2.3) |
| M+ | 7 (15.9) |
| Primary vs Salvage LT   - Primary - Salvage | 20 (45.5)  24 (54.5) |
| Graft type   - Whole - Partial | 30 (68.2)  14 (31.8) |
| Donor type   - Living - Deceased | 10 (22.7)  34 (77.3) |
| Sirolimus use   - Yes - No | 31 (70.5)  13 (29.5) |
| Bile duct anastomosis   - Duct-to-duct - Cholangioenteric | 27 (61.4)  17 (38.6) |
| GRWR (%) | 2.3 (0.7-5.7) |
| Intraoperative blood loss (ml) | 100 (50-1000) |
| Tumor recurrence   - Yes - No | 16 (36.4)  28 (63.6) |
| Death   - Yes - No | 5 (11.4)  39 (88.6) |

Abbreviations: PRETEXT, pretreatment extent of disease; V, hepatic vein tumor invasion; P, portal vein tumor invasion; E, extrahepatic disease contiguous with the main liver tumor; F, multifocal liver tumor; R, tumor rupture at diagnosis; M, distal metastasis; AFP, α-fetoprotein; LT, liver transplantation; GRWR, graft-to-recipient weight ratio.
